# Confinement-Controlled Rearrangements in Dioxolane Upgrading on H‑ZSM‑5 Revealed by Periodic DFT

**DOI:** 10.1021/acs.jpcc.6c01905

**Published:** 2026-07-09

**Authors:** Chenjiao Bu, Liangliang Huang, Michael J. Cordon, Andrew D. Sutton, Xiaokun Yang

**Affiliations:** † School of Sustainable Chemical, Biological & Materials Engineering, 6187 University of Oklahoma, Norman, Oklahoma 73019, United States; ‡ Manufacturing Science Division, 6146 Oak Ridge National Laboratory, Oak Ridge, Tennessee 37830, United States; § Chemistry Division, 5112 Los Alamos National Laboratory, Los Alamos, New Mexico 87544, United States

## Abstract

Zeolitic Brønsted acid sites catalyze carbocation rearrangements central to upgrading biomass-derived oxygenates. Here we elucidate the mechanism of dioxolane conversion to methyl ethyl ketone and isobutanal on H-ZSM-5 using periodic density functional theory on the MFI model, complemented by ab initio molecular dynamics to probe confinement effects. Dioxolane adsorption at the Brønsted site is followed by protonation-assisted ring opening to form an oxocarbenium intermediate stabilized by the deprotonated framework. From this common intermediate, selectivity is governed by two competing rearrangements, namely, the 1,2-hydride shift with a free-energy barrier of 18.05 kcal mol^–1^ at 498 K leading toward MEK, and the 1,2-methyl shift with a higher barrier of 25.40 kcal mol^–1^ leading toward isobutanal. The hydride-shift channel is kinetically preferred over the methyl-shift channel, lowering the isobutanal/MEK ratio below the 3:1 limit expected for equal branching. Adsorption thermodynamics further indicate stronger stabilization of MEK than isobutanal within ZSM-5 channels, suggesting that confinement-controlled binding can bias product distributions in addition to intrinsic rearrangement barriers. These results highlight how Brønsted acidity and pore confinement jointly shape the rearrangement landscape in MFI zeolites.

## Graphical abstract

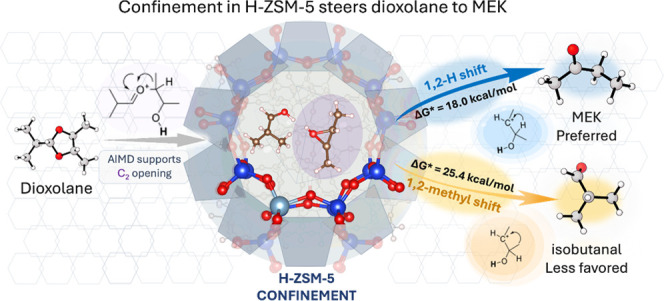

## 1 Introduction

Biomass-derived 2,3-butanediol (2,3-BDO) is an important C_4_ platform molecule for the sustainable production of fuels and chemicals.^1–3^ Upgrading routes that yield carbonyl products such as methyl ethyl ketone (MEK) and isobutanal are particularly attractive because MEK is a versatile solvent and fuel blend stock, while isobutanal is a valuable intermediate for fine-chemical synthesis.^4,5^ However, direct acid-catalyzed conversion of 2,3-BDO is often complicated by its high boiling point and strong hydrophilicity, which hinder separation and make reaction control in water-rich environments challenging.^6–8^ A practical alternative is an acetal-mediated, reactive-separation strategy in which 2,3-BDO is first converted with an aldehyde to a dioxolane, followed by zeolite-catalyzed cracking/rearrangement of the dioxolane to produce MEK and isobutanal.^6,9^ Among solid Brønsted acids evaluated for these transformations, H-ZSM-5 (MFI) provides a well-defined parent Brønsted-acid framework for probing how intrinsic acidity and pore confinement steer carbocation rearrangements and product selectivity in dioxolane conversion.

Under Brønsted-acid catalysis, upgrading of 2,3-BDO and related acetal intermediates typically yields MEK and isobutanal, consistent with a pinacol-type rearrangement proceeding through oxocarbenium/carbocation chemistry.^10,11^ Across solid acids, these two carbonyls are commonly the dominant products.^12,13^ For H-ZSM-5, isobutanal is often reported in excess, with isobutanal/MEK molar ratios on the order of 1.5 ∼ 2.0.^6^ In the dioxolane cracking network considered here, ring opening generates one isobutanal together with a confined oxocarbenium intermediate. The subsequent rearrangement then competes between a 1,2-hydride shift that produces MEK and a 1,2-methyl (Wagner–Meerwein) shift that produces a second isobutanal. If these rearrangements occurred with equal probability, the expected overall isobutanal/MEK ratio would be 3:1, whereas the consistently smaller experimental ratios imply that the hydride-shift channel is kinetically favored under zeolite confinement. This interpretation is further consistent with reports that selectivity on H-ZSM-5 shifts with acid strength, with weaker Brønsted acidity favoring MEK and stronger acidity favoring isobutanal.^6^ Together, these observations motivate a detailed theoretical assessment of the competing rearrangement pathways and how the zeolite environment biases their free-energy barriers.

Despite extensive experimental investigations, the molecular origin of the MEK/isobutanal product distribution on H-ZSM-5 remains unresolved. In particular, the relative importance of the competing 1,2-hydride and 1,2-methyl shifts under zeolite confinement has not been established under catalytically relevant conditions. Prior theoretical studies have often relied on truncated cluster representations of zeolite active sites, which can provide useful local chemistry but offer only a limited description of the extended framework environment, including long-range electrostatics and steric confinement.^14,15^ As a result, predicted barriers for ring opening and subsequent rearrangements can be sensitive to model choice, obscuring how the MFI pore architecture and Brønsted acidity reshape the free-energy landscape that governs selectivity in H-ZSM-5.

Here we employ periodic density functional theory on a 96 T MFI model,^16–18^ complemented by ab initio molecular dynamics to probe the stability of key confined intermediates to elucidate the mechanism of dioxolane conversion over H-ZSM-5 and to rationalize selectivity toward MEK versus isobutanal in 2,3-BDO-derived acetal upgrading. We map the elementary steps comprising adsorption, protonation, C2-selective ring opening, and the competing 1,2-hydride and 1,2-methyl shifts by locating transition states and computing corresponding free-energy barriers at 498 K. The resulting free-energy landscape identifies hydride migration as kinetically preferred over methyl migration under confinement, consistent with experimentally observed ratios below the 3:1 limit expected for equal branching. In addition, adsorption thermodynamics indicate stronger stabilization of MEK than isobutanal within MFI channels, implying that product binding can further bias distributions beyond intrinsic rearrangement kinetics. Together, these results establish a molecular picture of how Brønsted acidity and pore confinement jointly control rearrangement pathways in H-ZSM-5, providing a mechanistic basis for interpreting and ultimately tuning selectivity in acetal upgrading.

## 2 Methods and Computational Details

### 2.1 Zeolite Models

Brønsted acid sites in aluminosilicate zeolites correspond to bridging hydroxyl groups, Si–O­(H)–Al, formed when a framework Si atom is substituted by Al and the resulting negative framework charge is compensated by a proton.^19^ The mixed covalent/ionic character of the Si–O­(H)–Al linkage and the geometric constraints imposed by the rigid zeolite lattice weaken the O–H bond, giving rise to pronounced Brønsted acidity.^20^ In this work, the Brønsted site in H-ZSM-5 was constructed by substituting Al at the crystallographic T12 position of the MFI framework^21,22^ and protonating the adjacent framework oxygen O20, yielding the T12–O20 bridging hydroxyl located at a channel intersection (Figure 1a). Throughout, T labels tetrahedral framework positions (Si/Al) and O labels framework oxygen indices. The site choice follows the configuration analyzed by Opalka and Zhu,^22^ who reported that the intersection T12–O20 site exhibits relatively strong acidity compared with other MFI sites (e.g., T6), consistent with a larger Si–O­(H)–Al angle and a longer O–H bond. More broadly, the T12–O20 model should be regarded as a site-specific representation of an accessible Brønsted acid site at the MFI channel intersection, rather than a universal description of all acid-site environments in real H-ZSM-5. The MFI framework contains 12 crystallographically distinct T-sites, and previous experimental and computational studies have shown that framework Al siting is synthesis-dependent and can influence acid-site accessibility, local confinement, catalytic activity, and selectivity.^23,24^ Nevertheless, the T12 intersection site is a commonly used and chemically meaningful model in periodic H-ZSM-5 calculations, as it provides an accessible confined environment for adsorbates and transition states.^25,26^ A systematic PBE-D3 comparison of all 12 MFI T-sites by Smith et al. showed that transition-state energies vary with Al siting, while the commonly used T12 site gives among the lower barriers.^25^ Therefore, the free-energy barriers reported in this work should be interpreted as site-specific values for the representative T12–O20 intersection Brønsted site under low-coverage conditions.

**Figure 1:**
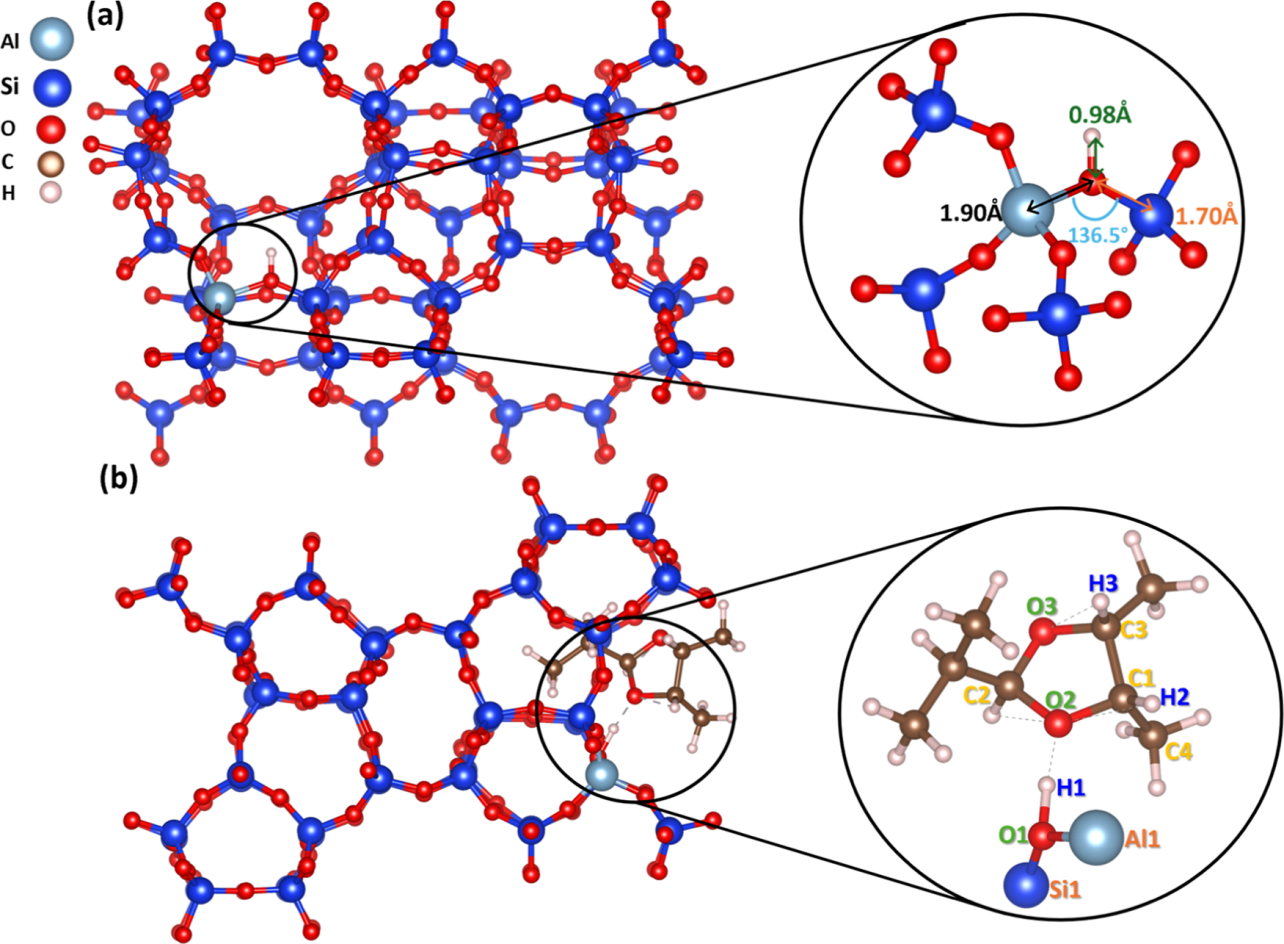
Periodic H-ZSM-5 model and dioxolane adsorption complex used in this work. (a) Periodic MFI unit cell with composition Si_95_Al_1_O_192_H_1_, illustrating the straight and sinusoidal channel system and a zoomed view of the Brønsted acid site formed by Al-substitution at T12 and protonation of the adjacent framework oxygen O20 (T12–O20) at a channel intersection. Selected optimized geometric parameters of the acid site are indicated (O–H = 0.98 Å; Si–O = 1.70 Å; Al–O = 1.90 Å; Si–O–Al = 136.5°), consistent with prior characterization of the T12 site.^19^ (b) Optimized adsorption configuration of dioxolane at the Brønsted site within the MFI pore; atom labels used in the mechanistic discussion are shown. Color code: Si (dark blue), Al (light blue), O (red), C (brown), H (white).

All periodic calculations in this study were performed on the conventional orthorhombic 96T MFI unit cell containing 96 tetrahedral atoms and 192 framework oxygen atoms, with composition Si_95_Al_1_O_192_H_1_ (Si/Al = 95).^27^ The Brønsted proton was placed on O20 adjacent to Al at T12, and the initial O–H vector was oriented toward the straight channel (Figure 1a). This periodic representation explicitly captures pore confinement and long-range electrostatics of the zeolite framework. An optimized adsorption complex of dioxolane at the Brønsted site and the atom labels used in the mechanistic discussion are shown in Figure 1b.

### 2.2 Computational Details

Periodic electronic structure calculations were performed using the Vienna ab initio Simulation Package (VASP, version 5.4.4.18Apr17).^28,29^ The projector augmented-wave (PAW) method was employed together with the Perdew–Burke–Ernzerhof (PBE) exchange–correlation functional within the generalized gradient approximation (GGA).^30–32^ A plane-wave kinetic-energy cutoff of 500 eV was used throughout. All calculations were carried out on the periodic H-ZSM-5 model described in Section 2.1, with lattice vectors fixed while allowing all atomic positions to relax.

Geometry optimizations were performed using the conjugate-gradient algorithm until the total energy change was below 1 × 10^–6^ eV and residual forces on each atom were below 0.01 eV Å^–1^. The Gaussian smearing of ISMEAR is 0 and SIGMA is 0.05 eV. Long-range dispersion interactions were included using the Grimme D3 scheme with Becke–Johnson damping, as implemented in VASP through IVDW = 12.^33,34^ Symmetry was disabled to ensure consistent force evaluation for low-symmetry adsorbate-zeolite configurations. Dipole corrections were not applied because the simulation cell is overall charge-neutral.

Born–Oppenheimer ab initio molecular dynamics (AIMD) was performed for selected adsorbed intermediates in the periodic model to assess their dynamic stability under reaction-relevant conditions. The AIMD simulations were carried out in the microcanonical ensemble using IBRION = 0 and SMASS = −3, with a time step of 1.0 fs. Initial velocities were assigned from a Maxwell–Boltzmann distribution at 500 K. To test the reproducibility of the observed relaxation behavior, multiple independent trajectories were propagated with different random seeds for both the C1- and C2-derived configurations. For each configuration, short trajectories of 1 ps were performed, and representative longer trajectories were extended to 3 ps. The Brillouin zone was sampled at the Γ point during AIMD, and the electronic self-consistent loop at each time step was converged to 1 × 10^–6^ eV. Dispersion interactions were included using the Grimme D3 correction with Becke–Johnson damping. These trajectories were used to assess the structural stability of key confined intermediates and their spontaneous relaxation behavior in the zeolite environment.

Reaction pathways were mapped using the climbing-image nudged elastic band (CI-NEB) method^35–37^ to obtain approximate saddle points, followed by refinement of the highest-energy image using the dimer method.^38^ NEB calculations employed 5–9 images depending on pathway length. The maximum force on each NEB image was converged to below 0.05 eV Å^–1^ prior to dimer refinement, and dimer searches were converged to below 0.02 eV Å^–1^. Geometry optimizations, static energies, CI-NEB and dimer refinements employed a Γ-centered 2 × 2 × 2 *k*-point mesh. Stationary points were characterized by finite-difference vibrational analyses to ensure that minima exhibited no imaginary frequencies, whereas transition states exhibited one and only one imaginary frequency associated with the reaction coordinate. The clean parent H-ZSM-5 framework was first optimized with all atoms relaxed to obtain the reference zeolite structure. For subsequent adsorption, intermediate, and transition-state optimizations, selective dynamics was applied to reduce computational cost while retaining the long-range periodic MFI environment. The adsorbed organic species, the Brønsted proton, and the framework atoms in the local acid-site region were allowed to relax, whereas framework atoms far from the reaction center were kept fixed. The relaxed local region was defined to include the atoms directly involved in proton transfer, C–O bond cleavage, and hydride/methyl rearrangement, together with neighboring framework atoms that interact with the adsorbate. As a convergence check, the hydride-transfer pathway was recalculated with an expanded relaxed region, which changed the NEB barrier by less than 0.01 eV. Gibbs free energies were evaluated as

$$G\left(T\right)=E_{elec}+ZPE+H\left(T\right)-TS\left(T\right)$$

where *E*_elec_ is the DFT total energy. For adsorbed states, thermodynamic corrections were obtained from finite-difference harmonic vibrational frequencies of the adsorbate species with the zeolite framework fixed. For gas-phase molecules, ideal-gas translational, rotational, and vibrational contributions were evaluated at the corresponding temperature and 1 atm. Thermodynamic corrections in the main text were evaluated at 498 K, with values at 448 and 548 K provided in Table S1. Adsorption free energies were calculated as

$$\Delta G_{ads}\left(T\right)=E_{zeolite+adsorbate}-E_{zeolite}-E_{adsorbate}+\Delta G_{corr}\left(T\right)$$

where

$$\Delta G_{corr}\left(T\right)=G_{vib}^{ads}\left(T\right)-G_{thermal}^{gas}\left(T\right)$$

The resulting Δ*G*_ads_(*T*) values for R1, P1, and P2 are summarized in Table S2. For each elementary step, activation and reaction free energies were calculated relative to the corresponding adsorbed reactant state

$$\begin{array}{l} \Delta G^{‡}=G_{TS}\left(T\right)-G_{R}\left(T\right)\\ \Delta G_{rxn}=G_{P}\left(T\right)-G_{R}\left(T\right) \end{array}$$

where *G*_R_, *G*_TS_, and *G*_P_ are the corrected free energies of the reactant, transition state, and product states, respectively.

## 3 Results and Discussion

### 3.1 Overview of the Reaction Network

Figure 2 summarizes the reaction network for dioxolane conversion to MEK and isobutanal on H-ZSM-5, obtained from periodic DFT calculations with the zeolite model described in Section 2.1. Dioxolane first adsorbs at the Brønsted acid site (ZOH) and is activated by protonation at the ether oxygen (R1), forming a protonated dioxolane complex (DOL-H^+^) stabilized by the zeolite conjugate base (ZO^–^). Subsequent C2-selective ring opening (R2) cleaves the five-membered acetal to generate one isobutanal molecule together with a confined oxocarbenium intermediate anchored near the acid site, shown in Figure 2a.

**Figure 2:**
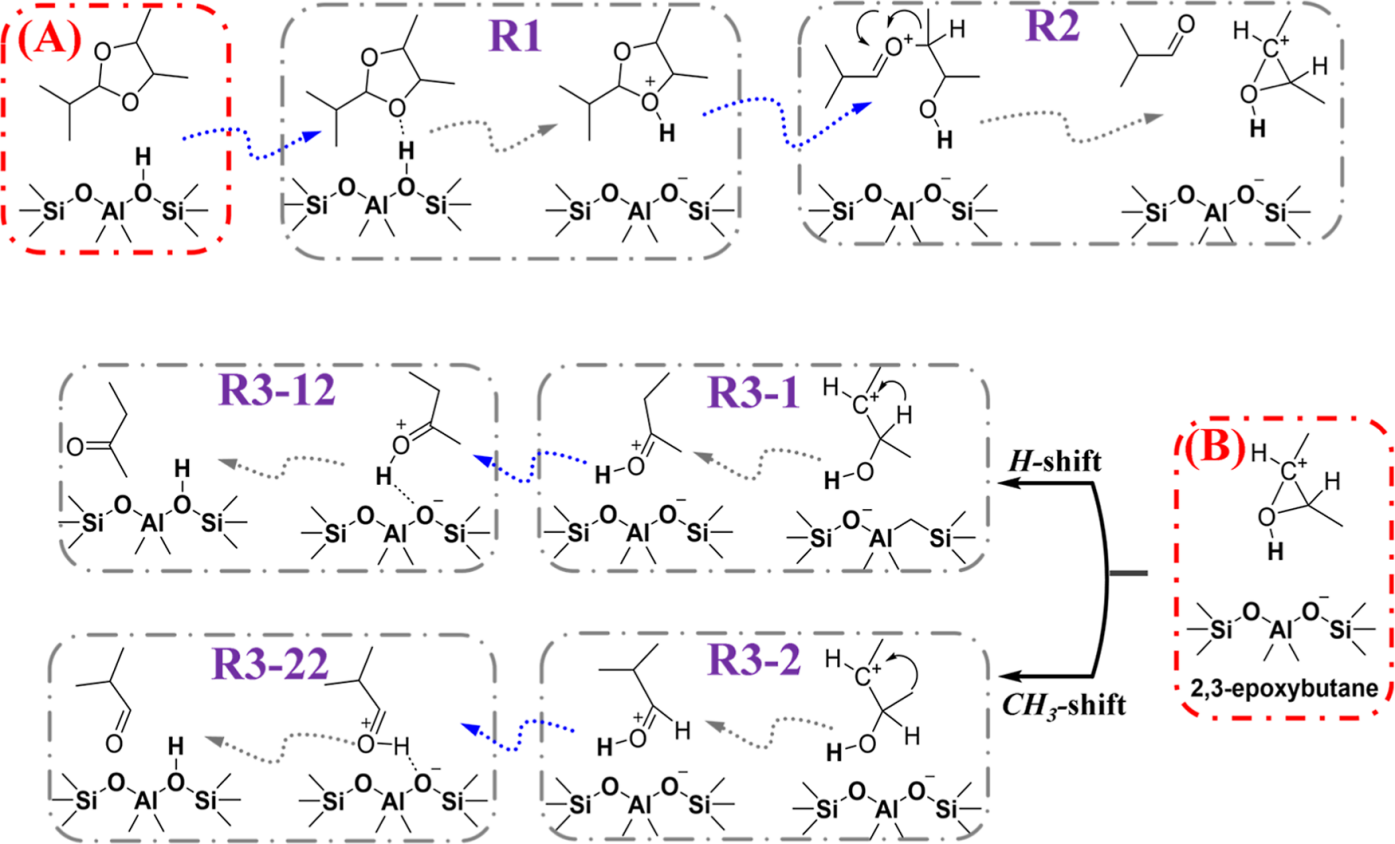
Reaction network for dioxolane conversion to MEK and isobutanal on H-ZSM-5. (a) Dioxolane adsorbs at the Brønsted acid site (ZOH) and is protonated at the ether oxygen (R1) to form DOL-H^+^ stabilized by the conjugate base (ZO^–^). C2-selective ring opening (R2) generates one isobutanal molecule and a confined oxocarbenium intermediate near the acid site. (b) From this common intermediate, selectivity is governed by two competing rearrangements: (i) 1,2-hydride shift (R3-1) followed by deprotonation (R3-12) to regenerate ZOH and yield MEK (overall: MEK + isobutanal per dioxolane), and (ii) 1,2-methyl (Wagner–Meerwein) shift (R3-2) followed by deprotonation (R3-22) to regenerate ZOH and yield a second isobutanal (overall: two isobutanal per dioxolane). The difference in stoichiometry between these branches underpins the expected isobutanal/MEK ratio for a given branching probability.

From this common oxocarbenium intermediate, the reaction can follow two competing rearrangement pathways, which ultimately control product selectivity inside the zeolite pores. In the 1,2-hydride-shift pathway (R3-1), hydride migration from the adjacent carbon to the cationic center produces a protonated MEK species. The deprotonation (R3-12) then regenerates ZOH and yields MEK in its adsorbed/zeolite-bound form. In the competing 1,2-methyl-shift pathway (R3-2), methyl migration produces a protonated isobutanal intermediate that undergoes deprotonation (R3-22) to regenerate ZOH and yield a second isobutanal. Thus, the hydride branch yields an overall stoichiometry of one MEK and one isobutanal per dioxolane, whereas the methyl branch yields two isobutanal per dioxolane and no MEK.

If the hydride and methyl rearrangements occurred with equal probability, the network in Figure 2b would predict an isobutanal/MEK ratio of 3:1. This is because the hydride pathway yields one MEK and one isobutanal, and the methyl pathway yields two isobutanal molecules. Experimentally, however, smaller ratios of about 1.5–2:1 are commonly observed on H-ZSM-5, implying that the hydride-shift channel is kinetically favored over the methyl-shift channel under catalytic conditions. The following subsections quantify this picture by reporting adsorption thermodynamics, transition-state structures, and free-energy barriers for each elementary step and by identifying how confinement within the MFI pore environment stabilizes key intermediates and transition states that bias selectivity.

### 3.2 Adsorption, Protonation and C2-Selective Ring Opening of Dioxolane

Section 3.1 highlights two mechanisms that connect directly to selectivity: (i) regioselective ring opening that establishes a common confined cationic intermediate, and (ii) competition between hydride and methyl rearrangements from that intermediate. Here in Section 3.2, we characterize the initial activation steps with respect to adsorption, protonation, and C–O scission, which generate the ring-opened intermediate from which the selectivity-determining rearrangements proceed according to eq 1 and Figure 2.

Initial activation of dioxolane on H-ZSM-5.Proton transfer from the Brønsted acid Site (ZOH) to dioxolane forms the protonated oxonium complex (DOL-H^+^) stabilized by the zeolite conjugate base (ZO^–^) (R1), followed by C2-selective ring opening that generates isobutanal and a confined cationic intermediate (R2).

1 $$\begin{array}{l} R1:\;ZOH+DOL\to TS1\to {ZO}^{-}+{DOL‐H}^{+}\\ R2:{DOL‐H}^{+}\to TS2\to isobutanal+{isobutylene\;oxide}^{+} \end{array}$$

In the adsorbed reactant complex, dioxolane is preorganized by hydrogen bonding to the Brønsted proton and by dispersive interactions with the pore walls, which together position the ether oxygen for proton transfer. In the initial adsorption complex, dioxolane is strongly stabilized inside the H-ZSM-5 pore, with an adsorption free energy of −18.05 kcal mol^–1^. The protonation step (R1) proceeds via a proton-transfer transition state (TS1) with a free-energy barrier of 24.40 kcal mol^–1^ relative to the initial adsorbed complex at 498 K (Figure 3). In TS1, the transferring proton is shared between the framework oxygen (O1) and the dioxolane oxygen (O2), with O1–H1 = 1.23 Å and H1–O2 = 1.19 Å. Formation of the protonated complex is thermodynamically favorable as Δ*G* is about −14.28 kcal mol^–1^ relative to the initial adsorption configuration. Additional adsorption configurations are shown in Figure S1. In addition, such process is accompanied by contraction of the newly formed O2–H1 bond to 1.08 Å and elongation of the adjacent C–O bond, thereby activating the acetal ring toward subsequent C–O scission.

**Figure 3:**
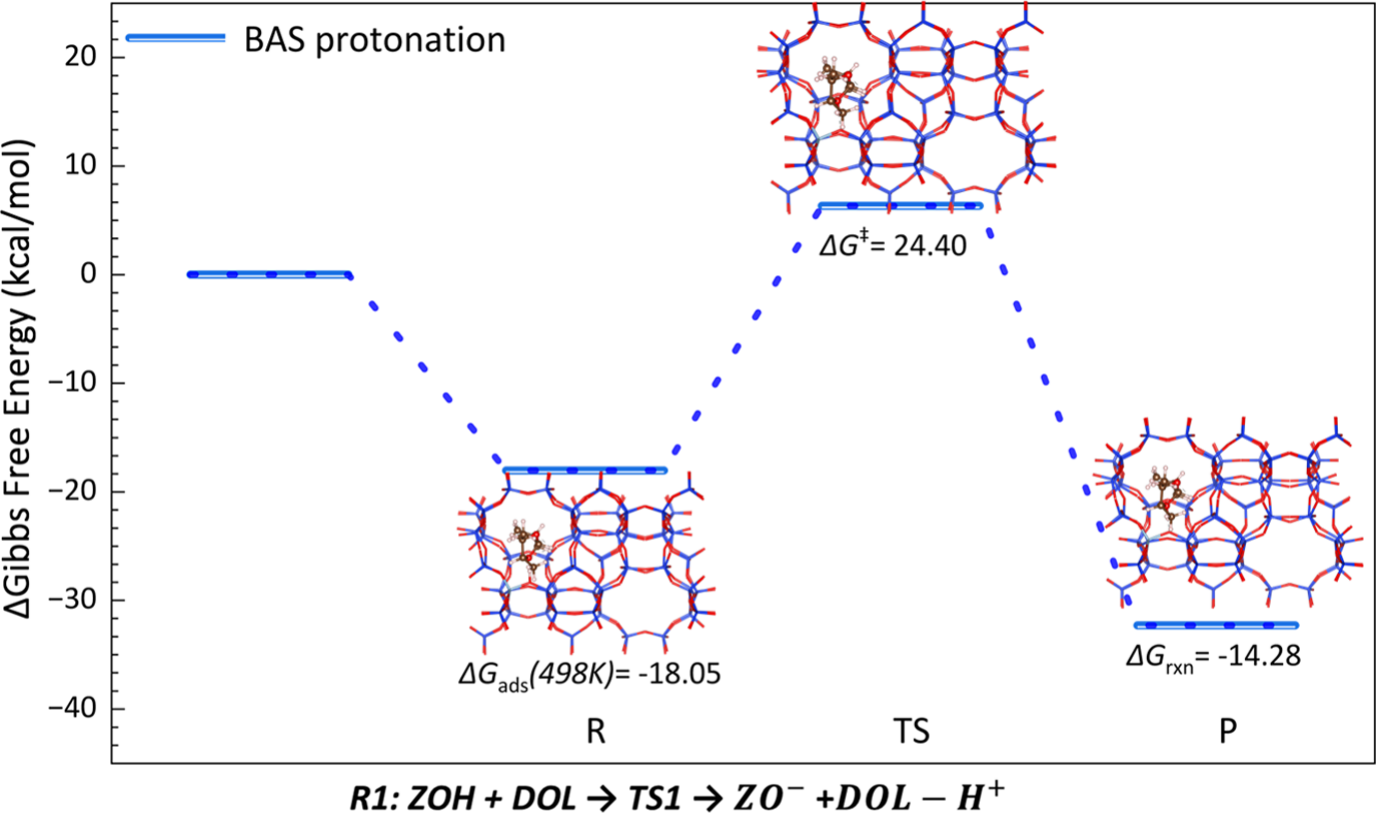
Protonation of dioxolane at the Brønsted acid site of H-ZSM-5. Free-energy diagram at 498 K for proton transfer from ZOH to the ether oxygen of dioxolane (R1), showing the adsorbed reactant complex, the proton-transfer transition state (TS1), and the stabilized protonated complex (DOL-H^+^/ZO^–^). The adsorbed dioxolane reactant is placed according to Δ*G*_ads_ (498 K), while Δ*G* ^‡^ and Δ*G*_rxn_ are reported relative to this adsorbed reactant state.

Ring opening can, in principle, occur through cleavage at either the C1 or C2 position (Figure 4a), leading to distinct cationic fragments and different downstream chemistry. Upon optimization, both trial scission motifs tend to reclose, showing that these simple cleaved structures are not stable end points for a direct C1/C2 transition-state comparison. However, the C2-derived structure retains the proton on the molecular fragment, whereas the C1-derived structure transfers it back to the zeolite framework (Figure S2). To probe the viability of C1 cleavage under confinement, we examined a C1-scission configuration using both an isolated-molecule optimization (Figure 4b) and periodic AIMD in 96T model (Figure 4d). In the isolated-molecule optimization, the initially prepared C1-cleaved structure spontaneously relaxes to a more stable C2-cleaved configuration, accompanied by a large energy decrease (∼89 kcal mol^–1^; Figure 4b), indicating that the cleaved state is intrinsically unstable. Consistently, a 2 ps AIMD trajectory of the isolated C1-cleaved species shows substantial reorganization of the C1–O2 bonding­(Figure 4c). This instability is even more mechanistically informative under H-ZSM-5 confinement. When the C1-cleaved configuration is placed inside the zeolite pore, AIMD shows spontaneous conversion toward a C2-cleaved structure (Figure 4d). Specifically, the O2–C1 distance remains short about 1.45 Å, whereas the O2–C2 distance elongates during the trajectory almost 4.8 Å, demonstrating that confinement funnels the structure toward the C2-cleavage channel. This behavior suggests that the apparent C1-cleaved state is dynamically unstable in the confined environment. In contrast, AIMD initiated from the C2-cleaved configuration exhibits productive ring opening followed by subsequent hydride and proton transfer events (Figure 4e). The elongation of the C3–H2 distance together with the shortening of the C1–H2 distance is consistent with intramolecular H transfer­(1,2-hydride shift pathway), while the evolution of the H1–O2 distance indicates proton return to the framework and regeneration of the Brønsted acid site. Independent trajectories from both C1- and C2-cleaved initial structures reproduce the same qualitative behavior, supporting the assignment of C2 scission as the dynamically accessible ring-opening mode under H-ZSM-5 confinement. Therefore, the subsequent reaction network was constructed from the C2-cleaved epoxide/oxocarbenium-like intermediate, which serves as the common precursor for the competing hydride- and methyl-shift pathways. These results demonstrate that C1 cleavage is not dynamically stable in H-ZSM-5 under the conditions modeled here. In contrast, C2 cleavage yields a stable ring-opened intermediate in the periodic environment, indicating that C2-selective opening is the relevant channel on H-ZSM-5 under the conditions modeled here.

**Figure 4:**
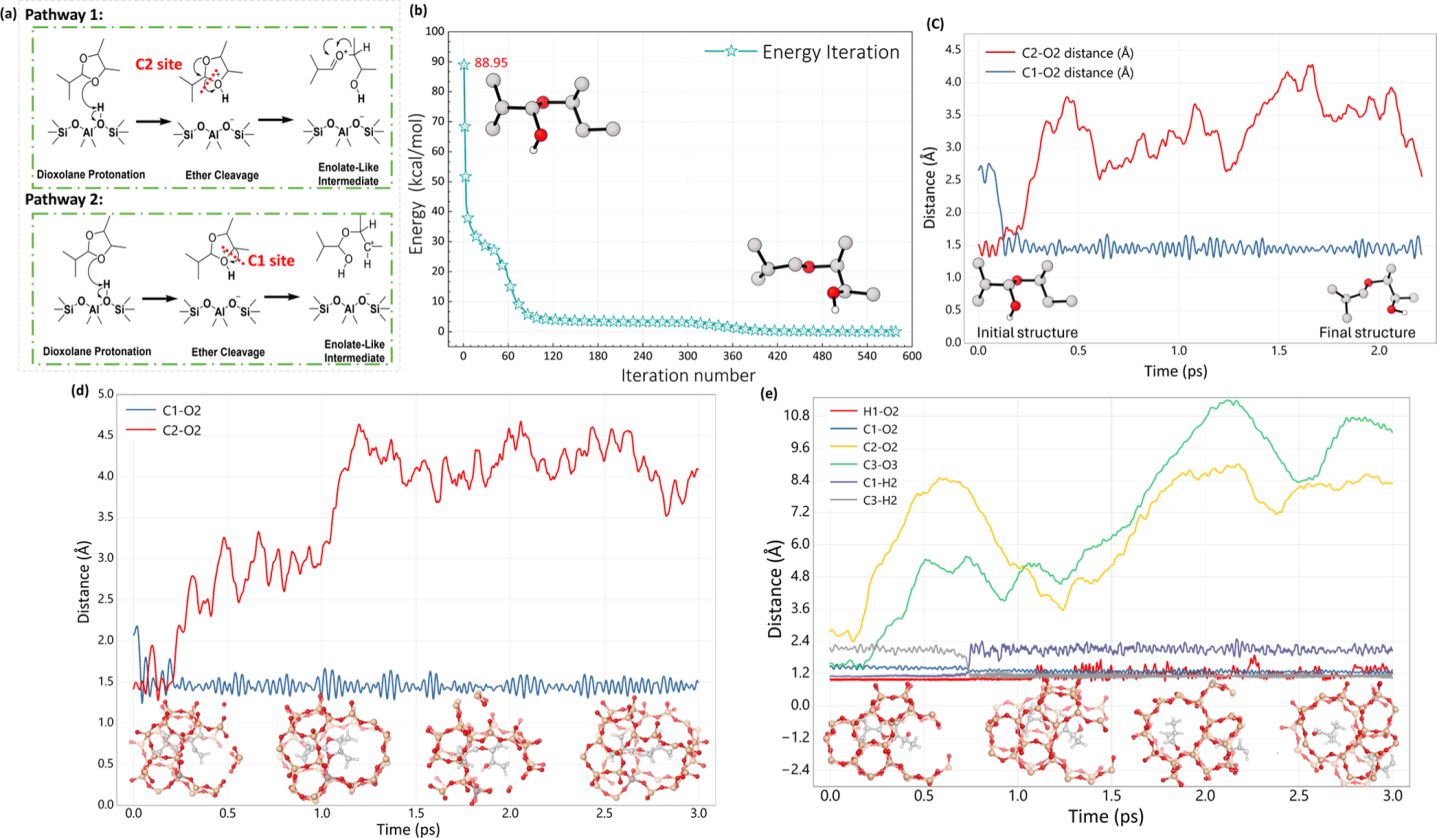
Regioselectivity of ether cleavage: C1 versus C2 ring opening under confinement. (a) Definition of the alternative C1- and C2-cleavage modes for C–O scission. (b) Potential-energy evolution during relaxation of a prepared C1-cleaved configuration, showing rapid downhill relaxation and structural reorganization. (c) AIMD trajectory of the isolated C1-cleaved species over 2 ps, monitored by the C1–O2 and C2–O2 distances, together with representative initial and final structures. (d) AIMD trajectory initiated from a C1-cleaved configuration inside H-ZSM-5, showing spontaneous conversion to a C2-cleaved configuration under confinement. (e) AIMD trajectory initiated from the C2-cleaved configuration in H-ZSM-5. Evolution of selected C–O, C–H, and O–H distances shows that C2 ring opening is followed by 1,2-hydride shift and proton return to the framework, leading to regeneration of the Brønsted acid site. Snapshots below the trajectories illustrate the corresponding structural evolution. All distances are reported in Å. Other independent AIMD calculations are provided in Figures S3–S6.

With C2 scission established as the viable pathway, we located the transition state for ring opening (TS2). As shown in Figure 5a, C2-selective ring opening (R2) proceeds with an activation free energy of 42.99 kcal mol^–1^ and yields products that are thermodynamically stabilized by −11.34 kcal mol^–1^ relative to the protonated reactant complex at 498 K. The ring-opening event produces isobutanal together with a confined cationic fragment that adopts an oxocarbenium-like geometry within the MFI pore environment. In the optimized product complex, this configuration is reflected by a short O2–C1 distance (1.26 Å) and a longer contact to the second carbon (O2–C3 = 3.08 Å). Upon removing the coadsorbed isobutanal and reoptimizing, the cationic fragment relaxes to a stable three-membered epoxide configuration with O2–C1 = 1.59 Å and O2–C3 = 1.56 Å, underscoring the stabilizing role of confinement and the conjugate base. The coproduced isobutanal molecule is located in the larger MFI pore region, where it remains separate from the confined cationic intermediate. Therefore, it was treated as a spectator species and excluded from the subsequent hydride- and methyl-shift calculations, as shown in Figure S7. Importantly, this ring-opened intermediate^39^ is the common branching point for the competing 1,2-hydride and 1,2-methyl shifts (Figure 5b). It is also worth noting that, although ring opening is the principal kinetic bottleneck in the computed network and serves as the rate-limiting early activation step, the relative barriers of the subsequent rearrangement steps control the MEK/isobutanal selectivity discussed in Sections 3.3 and 3.4.

**Figure 5:**
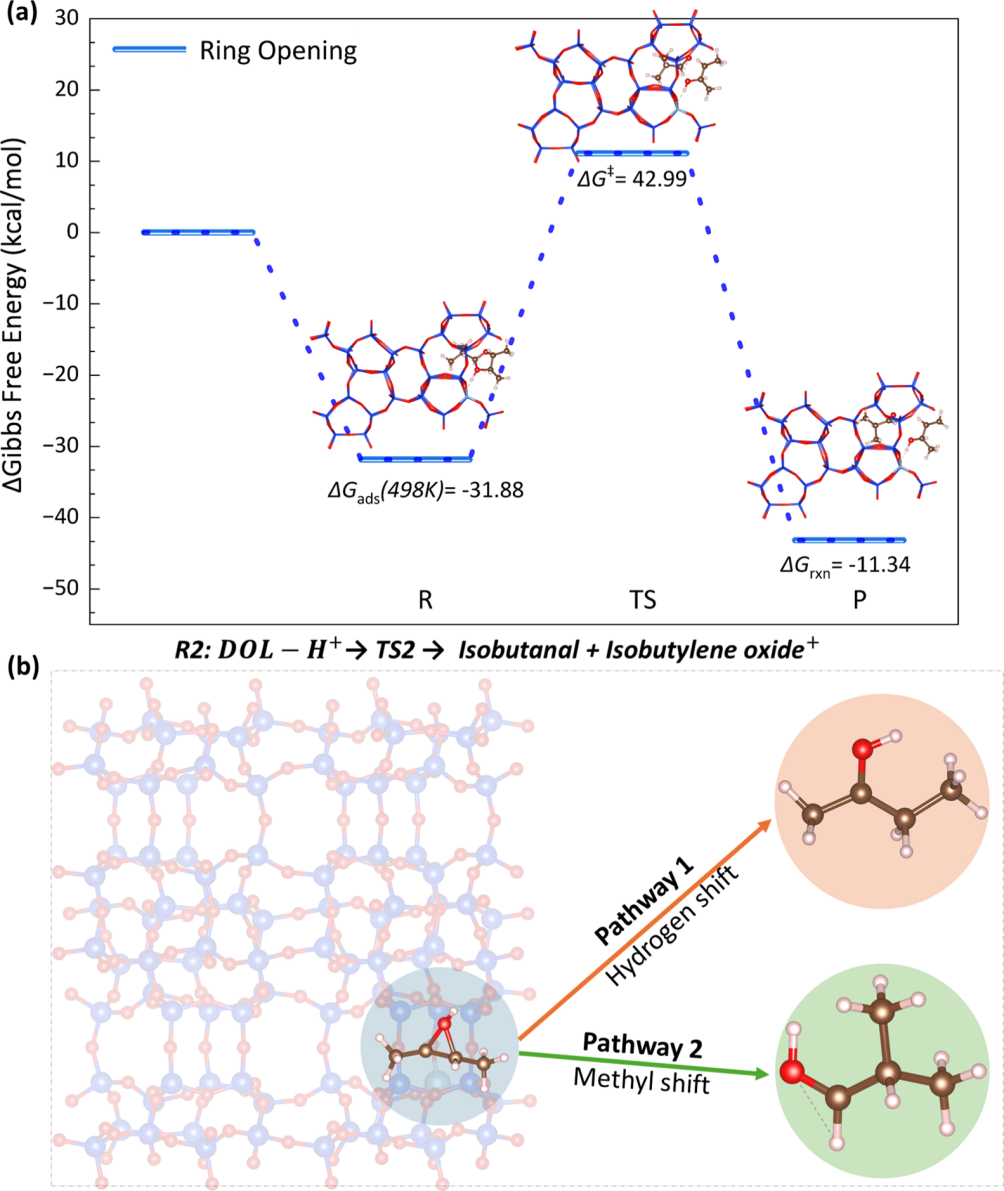
C2-selective ring opening of protonated dioxolane and formation of the branching intermediate. (a) Adsorption-based free-energy profile at 498 K for ring opening (R2) via TS2 to yield isobutanal and a confined cationic epoxide/oxocarbenium-like intermediate. The ring-opening reactant is placed according to its adsorption free energy, while Δ*G* ^‡^ and Δ*G*_rxn_ are reported relative to the protonated reactant complex. (b) Schematic of the subsequent competing rearrangements from this intermediate: 1,2-hydride shift (R3-1) and 1,2-methyl shift (R3-2).

### 3.3 1,2-Hydride Shift Reaction Pathway to Methyl Ethyl Ketone

Following the C2-selective ring opening in Section 3.2, the confined oxocarbenium-like intermediate can undergo two competing rearrangements that control the MEK/isobutanal product distribution: a 1,2-hydride shift (R3-1) and a 1,2-methyl shift (R3-2). Because the hydride branch yields one MEK together with the isobutanal already formed during ring opening (as shown in eq 2), whereas the methyl branch yields a second isobutanal, equal branching would imply an overall isobutanal/MEK ratio of 3:1. Reported values on H-ZSM-5 are typically smaller (∼1.5–2:1), consistent with a kinetic preference for the hydride-shift route under zeolite confinement.

Hydride-shift pathway to MEK from the ring-opened intermediate. (R3-1) 1,2-Hydride migration from the confined oxocarbenium-like intermediate forms a protonated MEK species; (R3-12) deprotonation transfers the proton back to the zeolite framework, regenerates ZOH, and yields neutral MEK.

2 $$\begin{array}{l} R3‐1:\;{isobutylene\;oxide}^{+}\to TS3‐1\to {MEK‐H}^{+}\\ R3‐12:\;{MEK‐H}^{+}+{ZO}^{-}\to TS3‐12\to ZOH+MEK \end{array}$$

As shown in Figure 6, the 1,2-hydride shift proceeds through TS3-1 with an activation free energy of 18.05 kcal mol^–1^ relative to the ring-opened rearrangement precursor at 498 K. The transformation is accompanied by clear signatures of hydride transfer and carbonyl reformation (Table 1). The donor C1–H2 bond elongates from the initial 1.10 Å to 1.66 Å at TS3-1 and to 2.06 Å in the product configuration, while the acceptor C3···H2 distance simultaneously contracts from the initial 2.18 Å to 1.20 Å at TS3-1 and to 1.11 Å in the product, confirming migration of H2 from C1 to C3. In parallel, the O2–C1 bond shortens from 1.59 Å to 1.29 Å, consistent with formation of a carbonyl-like C=O bond in the MEK framework. The O2–C3 distance increases from 1.56 Å to approximately 2.40 Å, indicating opening of the epoxide-like motif as the system reorganizes toward the protonated MEK intermediate.

**Figure 6:**
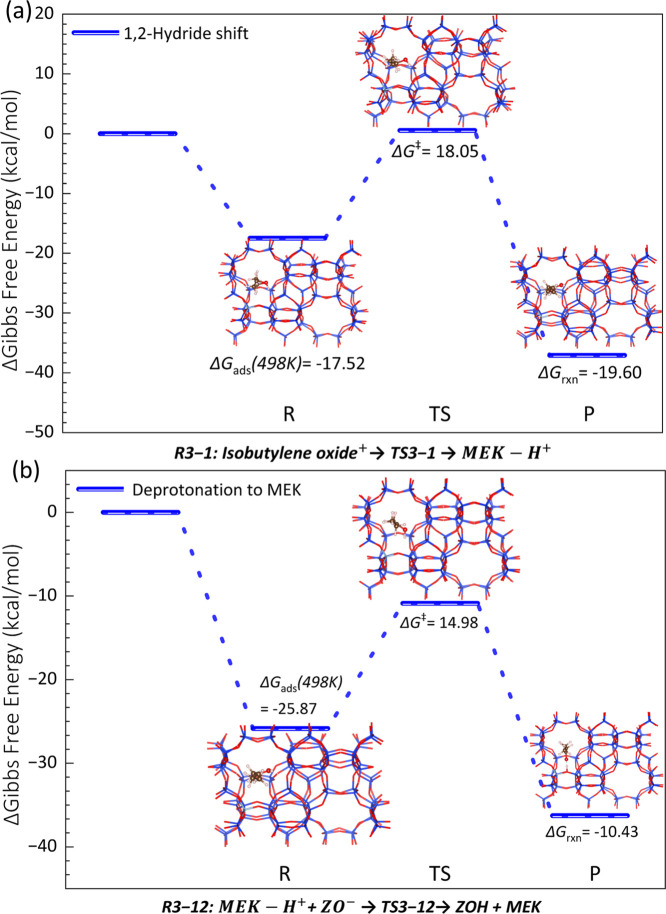
Free-energy profile for MEK formation via the 1,2-hydride-shift pathway. Adsorption-based Gibbs free-energy diagram at 498 K for (a) hydride migration (R3-1, TS3-1) from the ring-opened epoxide/oxocarbenium-like intermediate to a protonated MEK species, followed by (b) deprotonation (R3-12, TS3-12) to regenerate the Brønsted acid site (ZOH) and yield neutral MEK. The adsorbed reactant state in each panel is placed according to its adsorption free energy, while Δ*G* ^‡^ and Δ*G*_rxn_ are reported relative to the corresponding adsorbed reactant state. The hydride-shift and methyl-shift branching barriers are compared from the same ring-opened precursor.

**Table 1:** Distances between Key Atoms at Different Stages of Reaction Pathways for Hydride Shift, Methyl Shift

distance/Å	R3-1:1,2-hydride shift			R3-2:1,2-methyl shift		
	initial	TS3-1	final	initial	TS3-2	final
*d*_H2/C4–C1_	1.10	1.66	2.06	1.48	1.93	2.52
*d*_H2/C4–C3_	2.18	1.20	1.11	2.61	1.96	1.52
*d*_O2–C1_	1.59	1.30	1.29	1.59	1.31	1.28
*d*_O2–C3_	1.56	2.41	2.40	1.56	2.46	2.44

The protonated MEK intermediate subsequently undergoes deprotonation to regenerate the Brønsted acid site (ZOH) and yield neutral MEK (R3-12). The deprotonation transition state (TS3-12) corresponds to proton transfer from the adsorbed product back to a framework oxygen, restoring the Si–O­(H)–Al functionality required for catalytic turnover. Consistent with a proton-transfer event, the distance between the transferring proton and the accepting oxygen decreases substantially along the reaction coordinate (Table 2), while the donor O–H bond elongates in the product state. At 498 K, the 1,2-hydride shift is exergonic by 19.59 kcal mol^–1^. The subsequent deprotonation to MEK has a barrier of 14.98 kcal mol^–1^ and is further exergonic by 10.43 kcal mol^–1^ (Figure 6). The relatively low barrier for hydride migration provides a molecular basis for the experimentally observed preference toward MEK formation on H-ZSM-5 and for reported selectivity shifts with acid strength.^6^

**Table 2:** Selected Interatomic Distances Along the Deprotonation Pathways

distance/Å	R3-12: ZOH + MEK			R3-22: ZOH + isobutanal		
	initial	TS3-12	final	initial	TS3-22	final
*d*_H1–O1_	3.73	2.29	1.07	3.74	2.81	1.11
*d*_H1–O2_	1.07	0.99	1.42	1.00	1.00	1.36

### 3.4 1,2-Methyl Shift Reaction Pathway to A Second Isobutanal Molecule

In the alternative Wagner–Meerwein channel, the ring-opened oxocarbenium-like intermediate undergoes a 1,2-methyl migration (R3-2) that relocates the positive charge and yields a cationic product corresponding to protonated isobutanal (eq 3). Subsequent deprotonation (R3-22) transfers the proton back to the zeolite framework, regenerating the Brønsted acid site (ZOH) and producing an additional neutral isobutanal. Because C2-selective ring opening already generates one isobutanal (Section 3.2), this methyl-shift route results in an overall stoichiometry of two isobutanal molecules per dioxolane.

1,2-Methyl (Wagner–Meerwein) shift pathway to a second isobutanal molecule. (R3-2) 1,2-Methyl migration from the ring-opened epoxide/oxocarbenium-like intermediate forms a protonated isobutanal species; (R3-22) deprotonation transfers the proton back to the zeolite framework, regenerates ZOH, and yields an additional neutral isobutanal.

3 $$\begin{array}{l} R3‐2:\;{isobutylene\;oxide}^{+}\to TS3‐2\to {isobutanal‐H}^{+}\\ R3‐22:\;{isobutanal‐H}^{+}+{ZO}^{-}\to TS3‐22\to ZOH+isobutanal \end{array}$$

As shown in Figure 7, the 1,2-methyl shift proceeds through TS3-2 with an activation free energy of 25.40 kcal mol^–1^ relative to the rearrangement precursor at 498 K. This barrier is about 7.35 kcal mol^–1^ higher than the corresponding hydride-shift transition state (TS3-1, Section 3.3), indicating that methyl migration is substantially less competitive kinetically under confinement. Structurally, TS3-2 reflects a more asynchronous rearrangement than hydride transfer. The epoxide-like configuration opens as the O2–C3 interaction is cleaved, with O2–C3 elongating from 1.56 Å (reactant) to 2.46 Å in TS3-2 and final to 2.44 Å in the product (Table 1). In parallel, O2–C1 shortens from 1.59 Å to 1.31 Å at TS3-2, and 1.28 Å in the product, consistent with redistribution of positive charge toward the carbonyl-forming center as the carbon framework reorganizes. The methyl group itself migrates late along the reaction coordinate. The bond between the migrating methyl group and its original carbon elongates markedly from a typical C–C bond length of 1.48 Å to 1.93 Å in TS3-2 and 2.52 Å in the product. Meanwhile, the distance between the migrating methyl group and the acceptor carbon decreases from 2.61 Å to 1.96 Å at TS3-2 and reaches 1.52 Å in the product state (Table 1). This late approach and larger carbon-skeleton reorganization are consistent with the higher barrier for methyl migration relative to hydride transfer.

**Figure 7:**
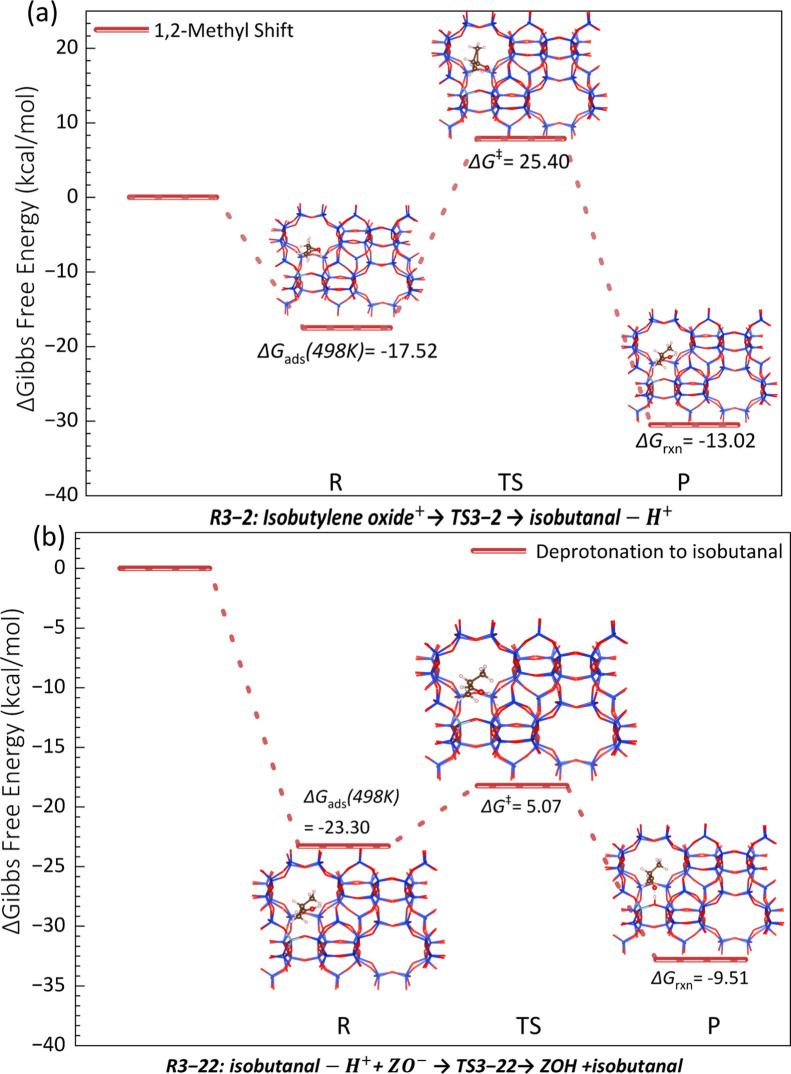
Free-energy profile for the 1,2-methyl shift pathway to isobutanal. Adsorption-based Gibbs free-energy diagram at 498 K for (a) methyl migration (R3-2, TS3-2) from the ring-opened epoxide/oxocarbenium-like intermediate to a protonated isobutanal species, followed by (b) deprotonation (R3-22, TS3-22) to regenerate the Brønsted acid site (ZOH) and yield a second neutral isobutanal molecule. The adsorbed reactant state in each panel is placed according to its adsorption free energy, while Δ*G* ^‡^ and Δ*G*_rxn_ are reported relative to the corresponding adsorbed reactant state.

Following methyl migration, deprotonation (R3-22) forms the second isobutanal and restores the Brønsted site. The deprotonation barrier is 5.07 kcal mol^–1^, lower than that of the hydride-branch deprotonation (TS3-12, 14.98 kcal mol^–1^). Specifically, the H1···O1 distance decreases from 3.74 Å (reactant) to 2.81 Å at TS3-22 and to 1.11 Å in the product, while the O2–H1 bond correspondingly lengthens from 1.00 Å to 1.36 Å, confirming proton return to the framework. Overall, the higher barrier for methyl migration establishes the methyl-shift route as the minor rearrangement channel relative to hydride migration under the conditions modeled here. Nevertheless, it is the only pathway that generates a second isobutanal molecule and therefore provides a direct mechanistic lever for increasing the isobutanal fraction when accessible.

### 3.5 Overall Reaction Profile

Figure 8 and Table S1 compile the computed free-energy landscape for dioxolane conversion to MEK and isobutanal on H-ZSM-5. Both rearrangement branches from the common ring-opened intermediate are thermodynamically downhill, but they differ substantially in kinetic accessibility. In particular, the 1,2-hydride shift (TS3-1) has a significantly lower free-energy barrier (18.05 kcal mol^–1^ at 498 K) than the competing 1,2-methyl (Wagner–Meerwein) shift (TS3-2, 25.40 kcal mol^–1^), establishing hydride migration as the kinetically preferred branching event under confinement. Because C2-selective ring opening produces one isobutanal prior to rearrangement, preferential hydride migration increases the MEK fraction and lowers the overall isobutanal/MEK ratio relative to the 3:1 limit expected for equal branching between hydride and methyl channels.

**Figure 8:**
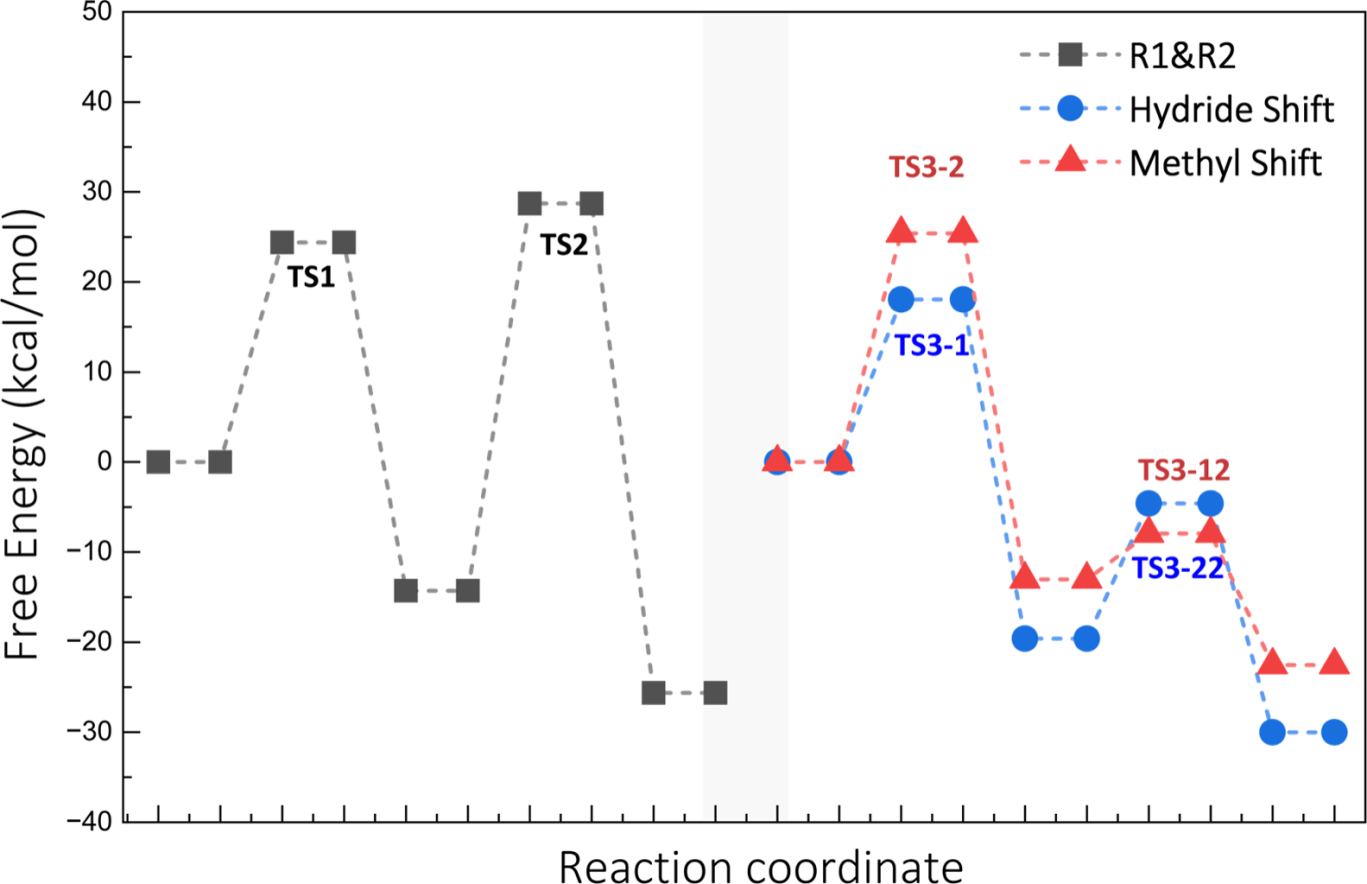
Overall free-energy profiles for MEK and isobutanal formation on H-ZSM-5. Computed Gibbs free-energy diagram at 498 K summarizing the elementary steps from the ring-opened intermediate through the competing rearrangement channels, highlighting the lower barrier for 1,2-hydride migration (TS3-1) relative to 1,2-methyl (Wagner–Meerwein) migration (TS3-2), which governs branching selectivity toward MEK versus isobutanal. R1 protonation and R2 ring opening are referenced to the adsorbed dioxolane complex, whereas the rearrangement pathways after the gray divider are referenced to the confined epoxide/oxocarbenium-like intermediate. The coproduced isobutanal from ring opening is treated as a spectator species.

Adsorption thermodynamics further indicate strong stabilization of products within the MFI pore environment (Table S2). The calculated adsorption free energies for dioxolane, isobutanal, and MEK are −18.05, −29.57, and −78.13 kcal mol^–1^, respectively, demonstrating markedly stronger binding for the carbonyl products, especially MEK, relative to the reactant. Such differential stabilization implies longer residence times and a greater propensity for readsorption of MEK at Brønsted sites, which can influence observed effluent product distributions in addition to intrinsic branching kinetics. Overall, the periodic free-energy profiles and adsorption energetics consistently support a mechanistic picture in which hydride migration dominates selectivity, while the methyl-shift pathway remains the only route to a second isobutanal molecule and therefore provides a direct lever for increasing the isobutanal fraction when the corresponding barrier becomes accessible.

## 4 Conclusions

Periodic density functional theory calculations on H-ZSM-5 were used to elucidate the elementary steps governing dioxolane conversion to MEK and isobutanal on a Brønsted acid site. The mechanism proceeds via adsorption and protonation of dioxolane to form a framework-stabilized oxonium/oxocarbenium complex (Δ*G* ^‡^ = 24.40 kcal mol^–1^ at 498 K), followed by C2-selective ring opening (TS2, Δ*G* ^‡^ = 42.99 kcal mol^–1^) that produces one isobutanal molecule together with a confined cationic intermediate. Ab initio molecular dynamics supports this regioselectivity by indicating that a putative C1-cleavage configuration is dynamically unstable under confinement, whereas C2-cleavage leads to a persistent ring-opened reactive intermediate.

From this common ring-opened intermediate, two rearrangements compete to govern product selectivity. The 1,2-hydride shift exhibits a lower free-energy barrier (Δ*G* ^‡^ = 18.05 kcal mol^–1^) than the competing 1,2-methyl (Wagner–Meerwein) shift (Δ*G* ^‡^ = 25.40 kcal mol^–1^), providing a molecular basis for why experimentally measured isobutanal/MEK ratios are typically below the 3:1 limit expected for equal branching between hydride and methyl channels. Adsorption thermodynamics further indicate stronger stabilization of MEK than isobutanal within the MFI pore environment, implying that confinement-controlled binding can bias observed product distributions in addition to intrinsic rearrangement kinetics. Overall, these results show that Brønsted acidity and pore confinement jointly shape the rearrangement landscape in H-ZSM-5, offering a mechanistic framework for interpreting and ultimately tuning MEK/isobutanal selectivity in upgrading 2,3-BDO-derived acetals.

The present analysis focuses on a single representative intersection Brønsted site in MFI under low-coverage conditions. While quantitative free energies can be sensitive to site location, coadsorbates (e.g., water), and the treatment of low-frequency modes in confined environments, the central conclusions follow from the relative barrier ordering and consistent trends obtained across the computed pathway. Building on this foundation, future work should quantify how Al siting and local environment (intersection vs channel sites, isolated Al vs paired Al) modulate the hydride- versus methyl-shift competition, and how coadsorbed water or higher coverages reshape protonation and ring-opening kinetics. Coupling the periodic free-energy landscape with microkinetic modeling, including adsorption/desorption and possible readsorption, would enable direct prediction of product ratios under experimentally relevant conditions, while extending the same mechanistic framework to other zeolite topologies would further clarify the role of confinement in steering rearrangement selectivity across solid acids.

## Supplementary Material





## Notes

Table S1: thermodynamic corrections in the main text were evaluated at 498 K, with values at 448 and 548 K. Table S2: the resulting Δ*G*_ads_(*T*) values for R1, P1, and P2. Figure S1: additional adsorption configurations. Figures S2–S6: other independent AIMD calculations. Figure S7: spectator species and excluded from the subsequent hydride- and methyl-shift calculations (PDF)
Additional computational details, optimized zeolite structures for the 96T periodic models (named zeolites structures/96T CONTCAR, R1&P1&P2_CONTCAR), VASP input files for geometry optimizations, transition-state searches, and AIMD simulations (VASP input/OPT input, VASP input/TS input and VASP input/AIMD input), and trajectory videos illustrating the key elementary steps on both models (reaction videos) (ZIP)
