# Supplementary material for: Confinement-Controlled Rearrangements in Dioxolane Upgrading on H‑ZSM‑5 Revealed by Periodic DFT
Source: J Phys Chem C Nanomater Interfaces. 2026 Jul 9;130(29):10219–30. doi: 10.1021/acs.jpcc.6c01905 (PMC13403309; doi:10.1021/acs.jpcc.6c01905)
Supplement: Supplementary file 1 [file jp6c01905_si_001.pdf]

Supporting Information for Publication:

Confinement-Controlled Rearrangements in Dioxolane Upgrading on H-ZSM-5 Revealed by Periodic DFT

Chenjiao Bu,[a] Liangliang Huang,[a*] Michael J. Cordon,[b] Andrew D. Sutton,[b] Xiaokun Yang[c*]

(a) School of Sustainable Chemical, Biological & Materials Engineering, University of Oklahoma, Norman, OK 73019, USA. Email: Chenjiao.Bu-1@ou.edu, HLL@ou.edu

(b) Manufacturing Science Division, Oak Ridge National Laboratory, Oak Ridge, Tennessee 37830, USA. Email: cordonmj@ornl.gov, suttonad@ornl.gov

(c) Chemistry Division, Los Alamos National Laboratory, Los Alamos NM 87544, USA. Email: xiaokuny@lanl.gov

(1) Figures

1. Adsorption structures of the 96T model

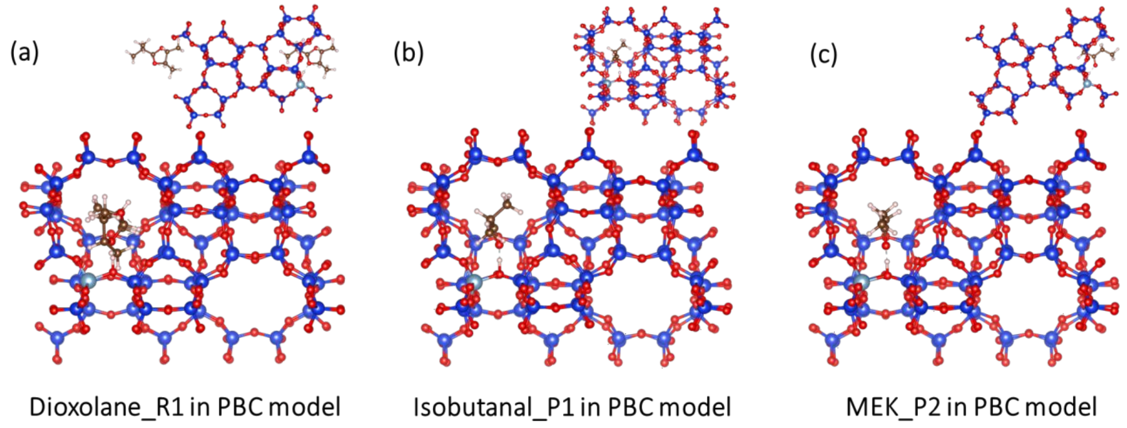

Figure S1. Optimized adsorption configurations of dioxolane and its main products within the 96T periodic model. The framework atoms are shown as Si (blue), O (red), and Al (light blue), with adsorbate atoms shown as C (brown) and H (white).

2. Analysis of ether cleavage between the C1 and C2 sites within the 96T model

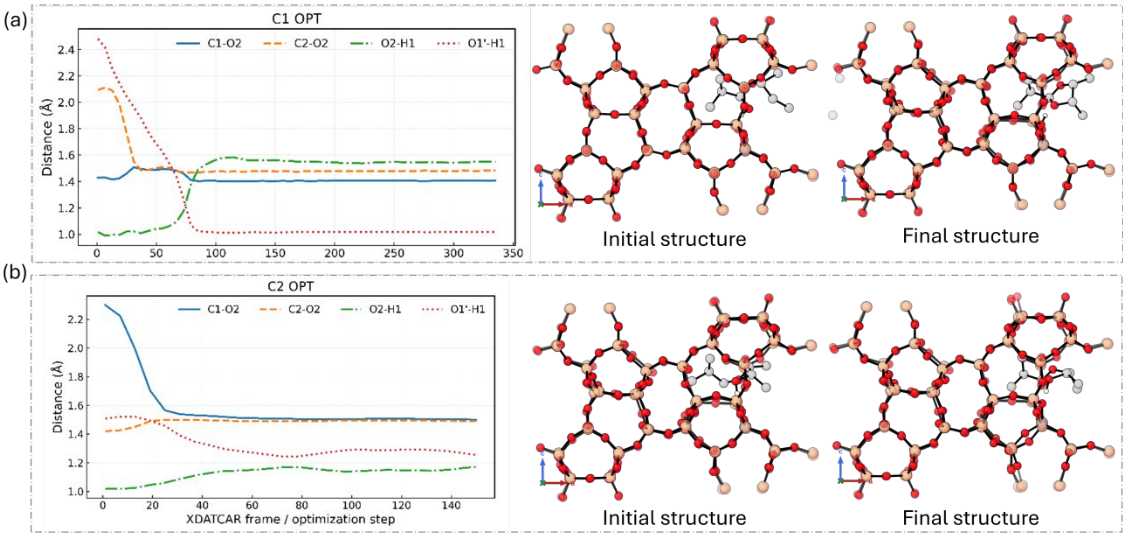

Figure S2 Structural relaxation of the C1- and C2-scission configurations. Left panels show the evolution of selected bond distances during geometry optimization, and the corresponding initial and final structures are shown on the right. For the C1-scission configuration, the initially elongated C2–O2 distance rapidly contracts to a normal C–O bond length, while the O2–H1 bond elongates and the O1–H1 bond shortens, indicating ring reclosure accompanied by proton transfer back to the framework Brønsted site. For the C2-scission configuration, the elongated C1–O2 distance also contracts during optimization, showing spontaneous ring reclosure; however, the proton remains associated with the molecular fragment rather than fully returning to the framework oxygen. These results indicate that isolated C1- or C2-scission structures are not stable minimum under unconstrained optimization.

3. AIMD trajectories from independent initial velocity seeds

To evaluate whether the ring-opened configurations are dynamically retained, we performed AIMD trajectories using different initial velocity seeds. The 3 ps AIMD trajectories for C1_seed_001 and C2_seed_004 are presented in the main text. Here, we summarize additional 1 ps AIMD trajectories obtained from independent initial velocity seeds to examine the reproducibility of the C1- and C2-cleavage dynamics. For the C1-cleaved trajectories, both C1_seed_002 and C1_seed_003 relax away from the C1-scission motif. In both cases, H1 remains associated with O2, O2–C1 stays near a bonded distance, and O2–C2 elongates during the trajectory. These results show that the C1-cleaved configuration is not dynamically retained and instead evolves toward a C2-scission-like structure.

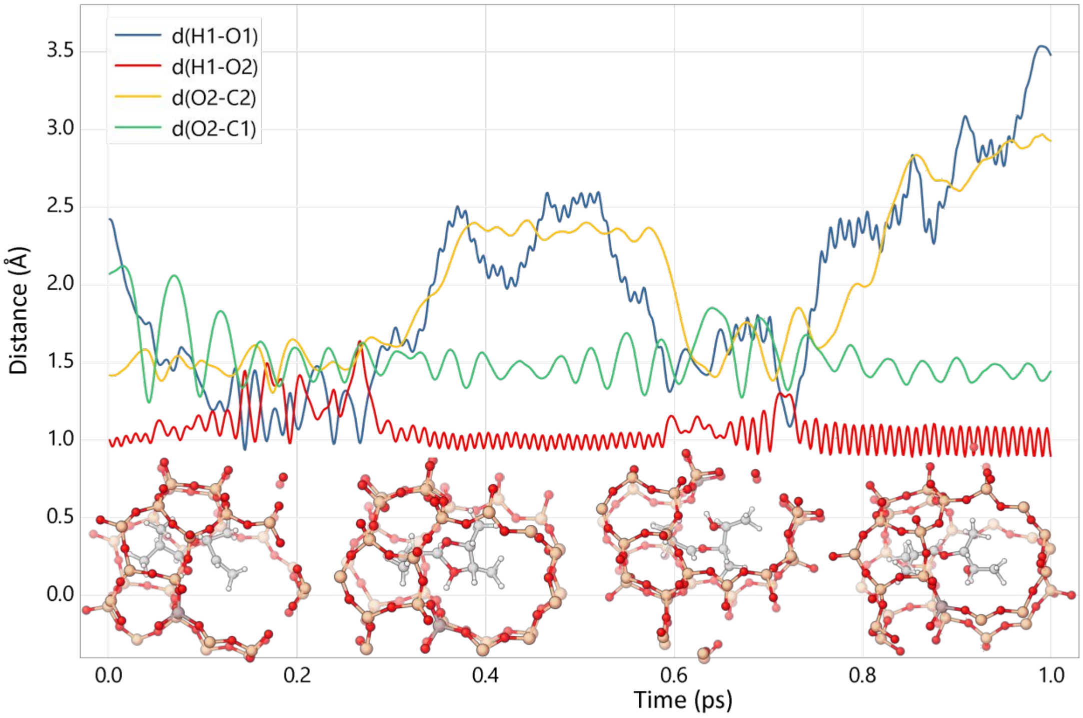

Figure S3. AIMD trajectory of C1_seed_002 starting from the C1-cleaved configuration. The selected bond distances show that the C1-scission motif is not preserved. H1 remains associated with O2, O2–C1 stays bonded, and O2–C2 elongates, indicating relaxation toward a C2-scission-like ring-opened structure. Representative snapshots along the trajectory show relaxation toward a C2-scission-like ring-opened structure.

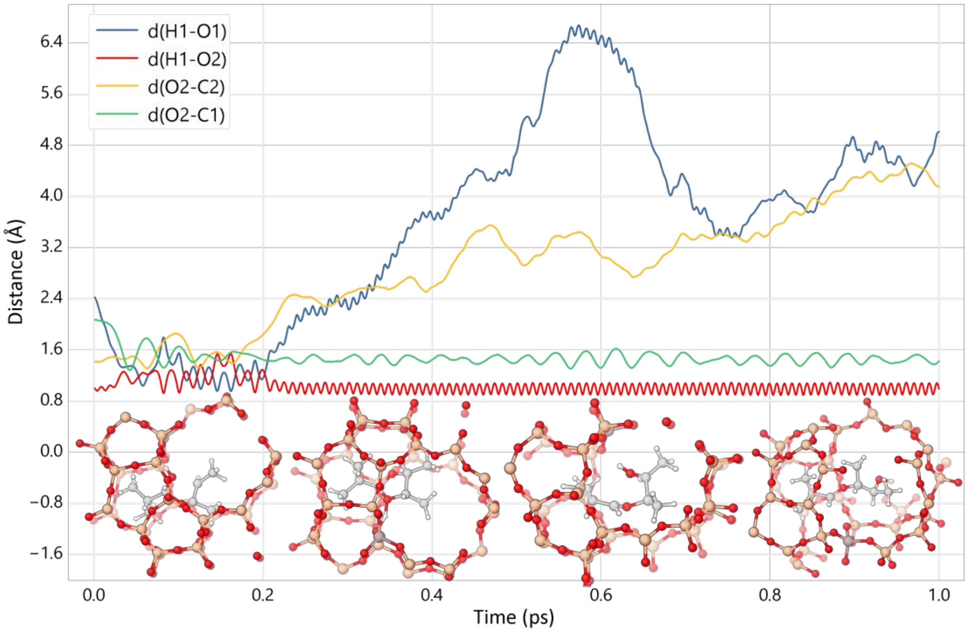

Figure S4. AIMD trajectory of C1_seed_003 starting from the C1-cleaved configuration. This independent trajectory shows the same qualitative behavior as C1_seed_002, with retention of the O2–C1 bond and elongation of O2–C2. The trajectory therefore further supports relaxation away from C1 scission toward a C2-scission-like structure.

For the C2-cleaved trajectories, both independent seeds retain C2-selective ring opening, but their subsequent evolution differs. In C2_seed_005, C2–O2 elongation is followed by a 1,2-hydride shift from C1 to C3, forming a hydride-shifted, MEK-like cationic intermediate. In C2_seed_006, C2–O2 elongation occurs without a completed 1,2-hydride shift, and the trajectory remains at the ring-opened stage, with the isobutanal molecule generated in R2 coexisting with a confined cationic epoxide/oxocarbenium-like intermediate.

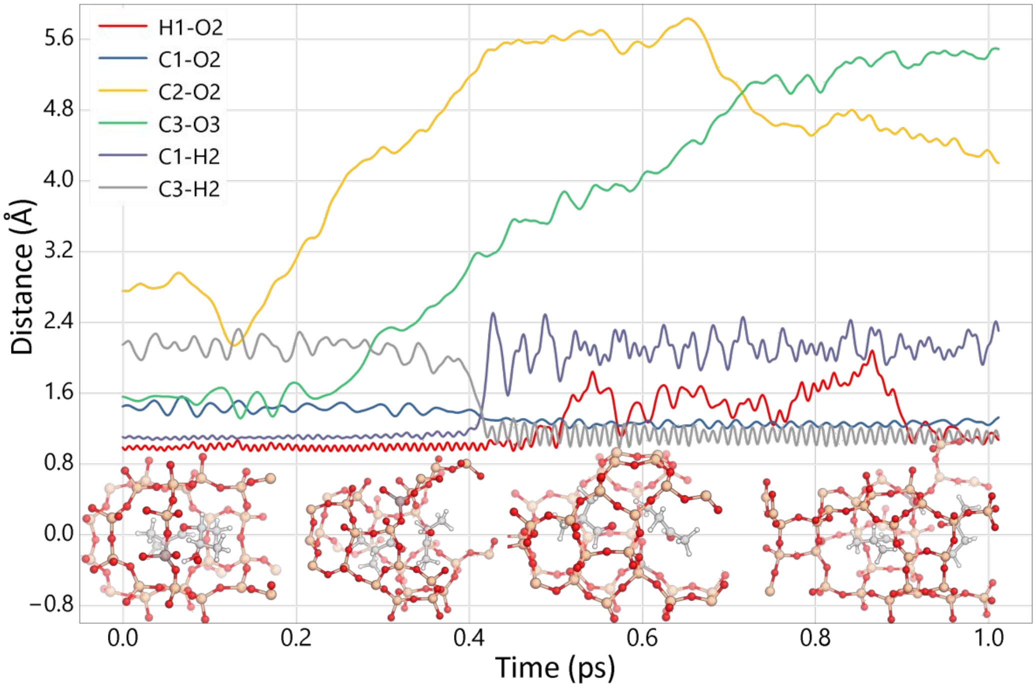

Figure S5. The AIMD trajectory of C2_seed_005 shows C2-selective ring opening, followed by 1,2-hydride transfer, as discussed in the main text. The C2–O2 bond elongates while C1–O2 remains short, confirming C2-selective cleavage. The subsequent elongation of C1–H2 and shortening of C3–H2 indicate H2 transfer from C1 to C3, giving a hydride-shifted, MEK-like cationic intermediate.

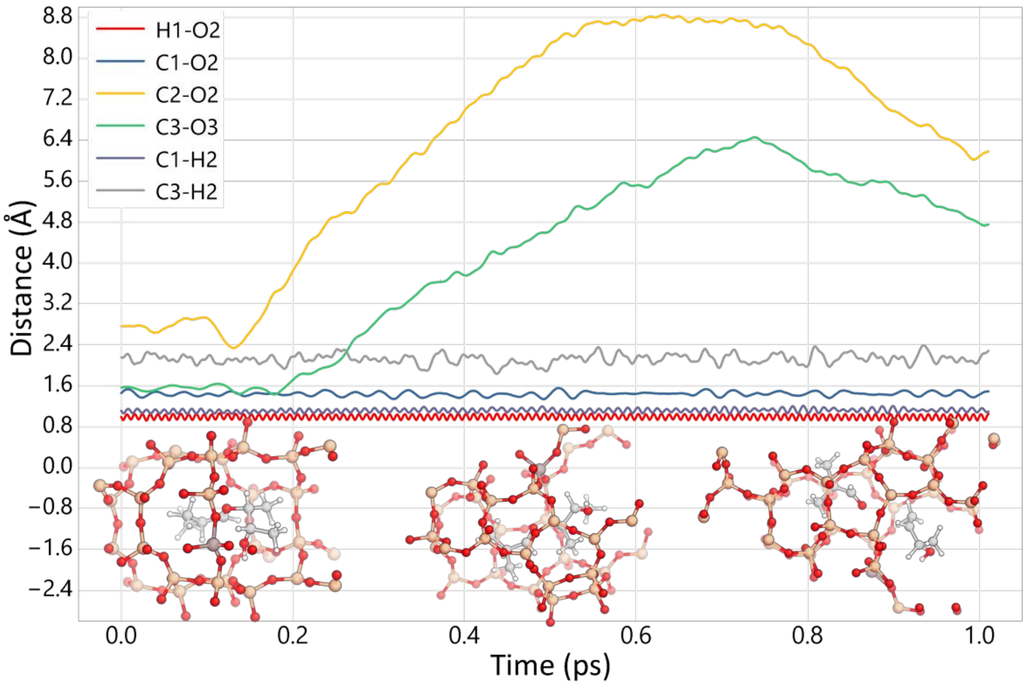

Figure S6. AIMD trajectory of C2_seed_006 showing C2-selective ring opening without complete hydride shift. The C2–O2 distance elongates while C1–O2 remains near a bonded distance, indicating C2-selective ring opening. The C1–H2 and C3–H2 distances do not show a persistent exchange, showing that hydride transfer is not completed within this trajectory.

Taken together, these independent AIMD trajectories show that C1-cleaved configurations are not dynamically retained. Instead, both C1 trajectories relax toward C2-scission-like structures. In contrast, the C2-cleaved trajectories consistently preserve C2-selective ring opening: C2_seed_005 proceeds to 1,2-hydride transfer and forms a hydride-shifted, MEK-like cationic intermediate, whereas C2_seed_006 remains at the ring-opened stage with an isobutanal molecule and a confined cationic epoxide/oxocarbenium-like intermediate.

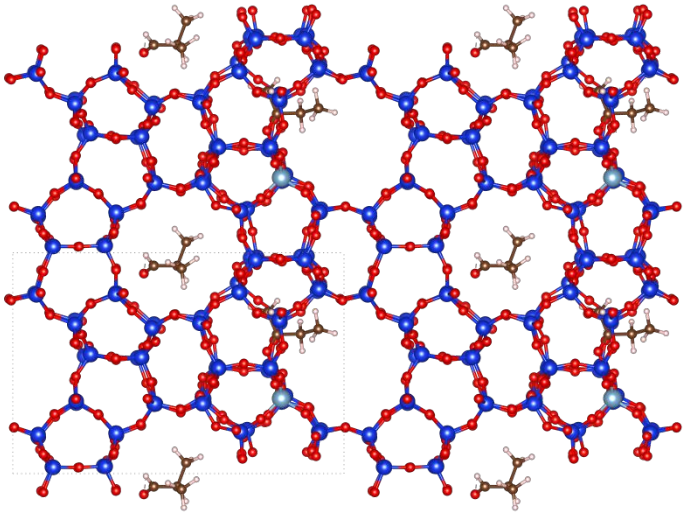

Figure S7. Periodic configuration after C2-selective ring opening. Following C2-selective ring opening, the first isobutanal molecule is accommodated within the enlarged pore region of the MFI framework, remaining spatially distinct from the confined cationic epoxide/oxocarbenium-like intermediate at the acid site. As the subsequent 1,2-hydride and 1,2-methyl shifts are local rearrangements of this cationic intermediate, the co-adsorbed isobutanal acts as a spectator species and is not included in the subsequent rearrangement calculations. This approach prevents the introduction of artificial crowding by a non-reactive product molecule under periodic boundary conditions, while retaining the local reactive intermediate responsible for MEK or the formation of the second isobutanal.

(2) Tables

Table S1. Gibbs free-energy barriers and reaction free energies for elementary steps in dioxolane conversion on H–ZSM–5 at experimentally relevant temperatures. All values are in kcal mol⁻¹ and were calculated using the vdW-corrected structures.

Step	Reaction description	448 K $\Delta G^{\ddagger}$	498 K $\Delta G^{\ddagger}$	548 K $\Delta G^{\ddagger}$	448 K ΔG_{rxn}	498 K ΔG_{rxn}	548 K ΔG_{rxn}
R1	Protonation of adsorbed dioxolane	24.43	24.39	24.34	−13.75	−14.28	−14.85
R2	C2-selective ring opening	42.97	42.99	43.03	−10.80	−11.34	−11.87
R3-1	1,2-hydride shift toward MEK	17.86	18.05	18.26	−20.14	−19.59	−19.02
R3-12	Deprotonation to MEK	15.16	14.98	14.80	−9.85	−10.43	−11.04
R3-2	1,2-methyl shift toward isobutanal	25.22	25.40	25.59	−13.01	−13.02	−13.04
R3-22	Deprotonation to isobutanal	5.03	5.07	5.13	−9.63	−9.51	−9.39

$Notes$: $\Delta G^{\ddagger} = G_{\mathrm{TS}} - G_{\mathrm{IS}}$; $\Delta G_{\mathrm{rxn}} = \sum G_{\mathrm{products}} - \sum G_{\mathrm{reactants}}$.

Table S2. Summary of temperature-dependent adsorption free energies, $\Delta G_{\mathrm{ads}}(T)$, in H–ZSM–5 (kcal mol⁻¹).

Adsorbate \ State Label	448 K	498 K	548 K
Dioxolane (R1)	−18.21	−18.05	−17.91
Isobutanal (P1)	−30.60	−29.57	−28.54
MEK (P2)	−78.20	−78.13	−78.12
